# Case Report-Severe Hyponatremia at Birth in a Premature Infant

**DOI:** 10.34763/jmotherandchild.20242801.d-24-00006

**Published:** 2024-10-23

**Authors:** Lujain Al-Omari, Adam Stranberg, Maria Franco Fuenmayor, Sunil Jain

**Affiliations:** The University of Texas Medical Branch at Galveston Galveston, TX UNITED STATES

## Abstract

**Background:**

Given the role of the placenta in maintaining maternal-fetal equilibrium, changes in maternal sodium levels affect the fetus. Clinicians must also account for the direct impact of maternal conditions and medications on the neonate. Gestational hyponatremia develops in approximately one-third of mothers with preeclampsia with severe features. Additionally, the use of selective antidiuretic (V2 receptor) agonist 1-deamino-8-D-arginine-vasopressin, commonly known as DDAVP, during pregnancy leads to maternal hyponatremia by inhibiting maternal diuresis. We present a case of severe hyponatremia in a premature infant born to a mother with preeclampsia with severe features who was taking DDAVP for von Willebrand Disease (VWD).

**Case:**

A preterm female infant was born at 34 weeks gestation to a mother with pre-eclampsia with severe features treated with magnesium sulfate, and the use of DDAVP for VWD was found to have severe hyponatremia (122 mmol/L). Causes of hyponatremia were explored, such as mineralocorticoid deficiency, renal tubular dysfunction, inappropriate secretion of antidiuretic hormone (SIADH), and renal failure. Initial investigation of the neonatal hyponatremia prompted obtaining a maternal serum sodium level, which also demonstrated severe hyponatremia (122 mmol/L), identical to the infant’s serum sodium level. The infant was managed with fluid restriction and close monitoring of serial serum and urine chemistries. Gradually, serum sodium levels increased and normalized by day 4 of life. We speculate that severe maternal hyponatremia induced by preeclampsia with severe features, along with the use of DDAVP during pregnancy, led to fetal and neonatal hyponatremia.

**Conclusion:**

DDAVP during pregnancy to treat VWD is associated with maternal hyponatremia and subsequent neonatal hyponatremia. It is important to monitor electrolytes in neonates born to mothers treated with DDAVP to promptly correct electrolyte abnormalities.

## Teaching points

– Maternal use of DDAVP is a cause of neonatal hyponatremia
– Maternal pre-eclampsia with severe features is also a cause of neonatal hyponatremia.
– The effect of DDAVP on fetal sodium levels is likely caused by the osmotic equilibration of sodium levels across the placenta.
– It is important to monitor electrolytes in neonates born to mothers with pre-eclampsia with severe features who are being treated with DDAVP during pregnancy to correct electrolyte abnormalities quickly.

## Introduction

Clinicians must consider the complex interplay between maternal and fetal sodium homeostasis that may result in direct complications for the neonate shortly after delivery. Given the role of the placenta in maintaining maternal-fetal equilibrium, changes in maternal sodium levels affect the fetus (1). While the fetus normally obtains 20–30 mL of water per day via bidirectional placental flow (2), maternal hyperosmolality has been shown to decrease amniotic fluid volume while hyper-hydration increases it (3). Maternal physiological hyponatremia occurs during pregnancy secondary to adaptation mechanisms of maternal water electrolytes homeostasis due to increased production of human chorionic gonadotropin, oxytocin and relaxin (2,3). Maternal hypothalamic osmoreceptors also reset to a new setpoint, which is triggered by decreased levels of plasma osmolality (4).

Clinicians must also account for the direct impact of maternal conditions and medications on the neonate. Gestational hyponatremia develops in approximately one-third of mothers with preeclampsia with severe features (5,6). Additionally, the use of selective antidiuretic (V2 receptor) agonist 1-deamino-8-D-arginine-vasopressin, commonly known as DDAVP, during pregnancy leads to maternal hyponatremia by inhibiting maternal diuresis (7). This effect of maternal DDAVP has been documented in both animal and human studies to induce fetal plasma hyponatremia, increased fetal urinary flow, and increased amniotic fluid volume (1,8).

We present a case of severe hyponatremia in a premature infant born to a mother with preeclampsia with severe features who was taking DDAVP for von Willebrand Disease (VWD).

## Case

A preterm female infant was born at 34 weeks gestation by elective cesarean section for pre-eclampsia with severe features treated with magnesium sulfate to a 25-year-old mother gravida 1 para 1 with VWD. Mother’s medical history was remarkable for heavy menstrual cycles, gingival bleeding and severe bleeding post appendectomy, resulting in diagnosis and administration of DDAVP and Factor VII/von Willebrand factor complex. She was unaware of what type of VWD she had and did not follow up with a hematologist after her initial consultation. She was unmedicated in the interim. During her hospitalization for delivery, the Hematology team was consulted, and she received one dose of DDAVP 20.24 mcg (0.3 mcg/kg), as well as 2,600 units of Factor VII/von Willebrand factor complex prior to neuraxial anesthesia to prevent bleeding. There were no bleeding complications during the mother’s hospitalization. The infant’s APGAR scores were 8 and 9 at 1 and 5 minutes of life, respectively. She weighed 2160 grams at birth, with a length of 47 cm and a head circumference of 32.5 cm. She was admitted to the Neonatal Intensive Care Unit (NICU) for prematurity and respiratory support.

A basic metabolic profile at 10 hours of life showed severe hyponatremia (122 mmol/L), prompting further investigation. The infant displayed no physical exam findings suggestive of hyponatremia, including neurologic manifestations. We explored possible causes of hyponatremia, such as mineralocorticoid deficiency, renal tubular dysfunction, inappropriate secretion of antidiuretic hormone (SIADH) and renal failure by checking urine osmolality, sodium and potassium levels. Urine osmolality was 109 mOsm/kg (normal range 50-1,100 mOsm/kg), urine sodium level was 16 mmol/L (normal < 20 mmol/L) and urine potassium level was 16.7 mmol/L (normal < 20 mmol/L). Serial urine chemistries remained normal, making mineralocorticoid deficiency unlikely. Normal serial serum urea (initial 5 mg/dL, normal 4–19mg/dL) and non-increasing creatinine (initial 0.65 mg/dL), along with normal urine output (1.8 to 2.9ml/kg/hr), ruled out renal failure and SIADH as possible etiologies.

**Figure 1.**
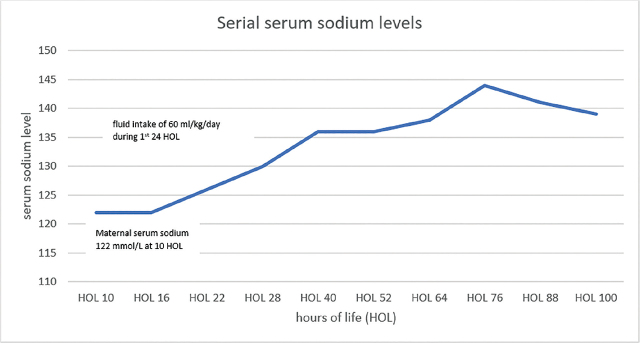
Trend of infant's sodium level at birth and after fluid restriction.

Initial investigation of the neonatal hyponatremia prompted obtaining a maternal serum sodium level, which also demonstrated severe hyponatremia (122 mmol/L), identical to the infant’s serum sodium level. Her BUN was 9 mg/dL (normal 7–23), and creatinine was 0.63 mg/dL (0.5–1.04), consistent with normal renal function. The infant was managed with fluid restriction and close monitoring of serial serum and urine chemistries. On day of life (DOL) 0, he was started on continuous dextrose infusion at 80 ml/kg/day with subsequent increase to 100 ml/kg/day on DOL 1. Fluid restriction to 60 ml/kg/day was implemented due to edema on physical exam. He was started on feeds at a total of 85 ml/kg/day. Gradually, serum sodium levels increased and normalized by day 4 of life (see figure 1). We speculate that maternal severe hyponatremia induced by preeclampsia with severe features, along with use of DDAVP during pregnancy, led to fetal and neonatal hyponatremia.

## Discussion

This is the first reported case of severe neonatal hyponatremia in a premature infant secondary to maternal preeclampsia with severe features and use of DDAVP during pregnancy. The complex interplay of sodium homeostasis for maternal and fetal well-being is modulated by the placenta, with known correlations between maternal and neonatal sodium levels.

Preeclampsia complicates 2–8% of pregnancies, and risk factors contributing to the pathology include pre-existing hypertension, insulin-dependent diabetes, previous preeclampsia, age greater than 40, pre-pregnancy obesity and family history of preeclampsia, among other risk factors (9). Complications of preeclampsia include maternal seizures and pulmonary edema, placental abruption, oligohydramnios and fetal growth restriction (9).

A recent review by Razavi et al. of 332 pregnancies complicated by preeclampsia with severe features found 9.7% were associated with hyponatremia (serum sodium <130 mEq/L) (10). Maternal hyponatremia equilibrates across the placenta, resulting in neonatal hyponatremia, as reported in infants born to mothers with preeclampsia (11).

Our literature review has revealed fetal hyponatremia induced by maternal DDAVP in animals (12,13), but no such reports were published on humans.

Von Willebrand disease (VWD) is a bleeding disorder caused by quantitative or qualitative defects of von Willebrand factor (VWF), an adhesive protein that binds platelets to the sub-endothelium and carries factor VIII (14). The severity of the disease varies across several types and subtypes, and DDAVP is a synthetic analog of the antidiuretic hormone vasopressin that is used to treat some types of VWD (14). DDAVP induces the release of VWF from endothelial cells, which prevents bleeding (15).

The use of DDAVP is safe during pregnancy, as it does not cross the placenta in detectable amounts (15,16). Ray et al. measured DDAVP concentrations in the maternal and fetal compartments of placental lobules and demonstrated that while at therapeutic maternal drug concentrations, DDAVP does not cross the placenta within detectable limits, but small amounts may cross the placenta at much higher drug concentrations (17). Therefore, hyponatremia in neonates born to mothers treated with DDAVP is more likely due to osmotic equilibration of electrolytes by the placenta rather than the direct effect of DDAVP on the fetus.

Sodium homeostasis shows significant changes during pregnancy secondary to increased arginine vasopressin (AVP) secretion from the posterior pituitary gland. The osmoregulatory action of AVP is mediated via V2 receptors located on renal collecting ducts. Stimulation of AVP V2 leads to water absorption in the collecting ducts (18). The use of DDAVP, in addition to the oxytocin and intravenous fluid that are commonly used during labor, could also increase the risk of hyponatremia (19). Based on the known pathophysiology and clinical presentation, we speculate that the severe neonatal hyponatremia in the present case of preterm infants may be due to preeclampsia with severe features along with maternal use of DDAVP for VWD.

Of note, a study published in 2013 by Jahromi et al. discusses that VWF deficiency could be a risk factor for preeclampsia, and DDAVP might prevent preeclampsia in women with VWF deficiency (20).

## Conclusion

We presented a case of hyponatremia in a preterm infant induced by maternal use of DDAVP in a mother with preeclampsia with severe features during pregnancy. The effect of DDAVP on fetal sodium level is likely caused by osmotic equilibration of sodium levels across the placenta. Our report emphasizes the importance of monitoring electrolytes in neonates born to mothers with pre-eclampsia with severe features, which may also cause neonatal hyponatremia, and who are treated with DDAVP during pregnancy, to identify and address electrolyte abnormalities promptly.
